# Chromosome evolution in *Iberolacerta*, a genus that deviates from the standard karyotype formula of Lacertidae

**DOI:** 10.1007/s10709-023-00194-w

**Published:** 2023-09-01

**Authors:** Horacio Naveira, Verónica Rojo, Iván Gómez-Seoane, Malcolm A. Ferguson-Smith, Jorge C. Pereira, Andrés Martínez-Lage

**Affiliations:** 1 Grupo de Investigación en Bioloxía Evolutiva, Departamento de Bioloxía, Facultade de Ciencias, CICA, Universidade da Coruña, A Coruña, Spain; 2 AllGenetics & Biology SL, A Coruña, Spain; 3 Grupo de Investigación en Terapia Celular y Medicina Regenerativa, INIBIC, Universidade da Coruña, A Coruña, Spain; 4 Department of Veterinary Medicine, Cambridge Resource Centre for Comparative Genomics, Cambridge, UK; 5 Animal and Veterinary Research Centre (CECAV), UTAD, AL 4AnimalS, Vila Real Vila Real, Portugal

**Keywords:** Reptilia, ZOO-FISH, iCGH, Sex-chromosomes, Microchromosome loss, W-autosome fusion, Satellite DNA

## Abstract

This paper describes the preparation of flow-sorted chromosome paints from the Iberian Rock lizard *Iberolacerta monticola*, exemplifying their subsequent use in cross-species comparisons of chromosome painting. We carried out comparative analyses of chromosome evolution in the congeneric species *I. galani* and *I. bonnali*, as well as in two other species of Lacertini (*Lacerta schreiberi* and *Timon lepidus*) whose sex chromosomes were also studied through comparative genomic hybridization. Most species of Lacertini possess a diplod number of 2n = 38, with 36 acrocentric macrochromosomes and 2 microchromosomes. However, the nine species included in the genus *Iberolacerta* do not possess microchromosomes. Furthermore, very conspicuous differences from the standard Lacertini karyotype were observed in the three Pyrenean species of this genus, which included several biarmed metacentrics and a Z_1_Z_2_W multiple sex-chromosome system. With the possible exception of *L. schreiberi*, all the species of the family Lacertidae described to date appear to share homologous Z chromosomes, which date back to the last common ancestor of the whole group. We provide conclusive evidence that *L. schreiberi* should no longer be considered an exception to this rule, and demonstrate that the loss of microchromosomes in *Iberolacerta* was produced by their fusion to a middle-sized chromosome. Furthermore, we show that the multiple sex-chromosome system of the Pyrenean species of *Iberolacerta* originated from the fusion of the ancestral W chromosome with one of the shortest autosomes, and provide additional evidence of the fast evolution of DNA sequences linked to the W chromosome in Lacertini.

**Supplementary Information:**

The online version contains supplementary material available at 10.1007/s10709-023-00194-w.

## Introduction

Evolution of chromosome number and chromosome morphology may seem of little significance in the current -omics era, given the many thousands of whole-genome sequencing projects (WGS) already fulfilled or underway. However, in Animals, only a small fraction of these projects have reached the chromosome assembly level (1979 out of 10,337 reports, NCBI Genome Database, last accessed 23/02/23). Paradoxically, in many respects, a similar situation was experienced during the first quarter of the 20th century, which was described by M.J.D. White as “the heyday of atomistic genetics.“ This period ended when the evolutionary importance of the physical basis of a species genetic system, i.e., its karyotype, was realized (White 1973).

Notwithstanding this apparent disregard for the study of karyotypes, there is a special kind of chromosomes whose evolution has received continuing attention over the years, namely animal sex-chromosomes. This is particularly so for squamate reptiles, since this group exhibits an astonishing diversity of sex-determining systems, which range from environmental sex determination to genotypic sex determination, including male heterogamety (XX/XY), female heterogamety (ZZ/ZW), and multiple sex chromosomes (Sarre et al. 2004; Pokorná and Kratochvíl 2009; Ezaz et al. 2010; Nielsen et al. 2020; Rovatsos et al. 2022). In reptiles, the study of sex-chromosomes during the last two decades has revealed that, contrary to previous claims of the overall high lability of their sex determination systems (Ezaz et al. 2010; Sarre et al. 2011; Matsubara 2019), and notwithstanding the independent origins of sex chromosomes in different families (Nielsen et al. 2020; Rovatsos et al. 2022), many groups possess old, long-term stable sex-chromosomes (Rovatsos et al. 2014a, b, 2015; Augstenová et al. 2021; Thépot 2021). Furthermore, recent studies have shown that relatively recent turnovers of sex-chromosomes are restricted to just a few genera or families (Gamble et al. 2015; Patawang et al. 2017; Sidhom et al. 2020; Augstenová et al. 2021; Keating et al. 2021). A possible bias in this respect cannot be excluded, since the available information on squamate sex-chromosomes is based on a relatively small fraction of the described species from this group (Mezzasalma et al. 2021). In any case, the Old World lizard family Lacertidae (Pyron et al. 2013) does not appear to be an exception to this pattern (Rovatsos et al. 2016), although the portrait of the evolution of their sex-chromosomes is still incomplete. With 370 species grouped into 45 genera (Uetz et al. 2022), the Lacertidae represents the predominant lizard group in Europe and a substantial component of the squamate reptile diversity in Africa (Arnold et al. 2007; Hipsley et al. 2009). The family, whose diversification started roughly 87 Mya, in the Late Cretaceous Epoch (Garcia-Porta et al. 2019), contains two subfamilies, Gallotinae and Lacertinae, with the latter composed of two monophyletic clades or *tribus* (after Arnold et al. 2007), the Lacertini, mainly Palearctic, and the Eremiadni, restricted to Africa. Genetic analyses indicate a fast diversification and radiation of Lacertini in the late Eocene, roughly 37 Mya (Hipsley et al. 2009; Garcia-Porta et al. 2019). Most species of Lacertini (and Eremiadni, for that matter) possess a diploid number of 2n = 38, with 36 acrocentric macrochromosomes and 2 microchromosomes (Arnold et al. 2007; Mezzasalma et al. 2021), with the most conspicuous exceptions to this karyotypic formula being the three Pyrenean species of the genus *Iberolacerta*, which include *I. bonnali*, *I. aranica* and *I. aurelioi*. The male karyotypes of these species consist of 2n = 24 *(I. bonnali*) and 2n = 26 (*I. aranica and I. aurelioi*) chromosomes. In addition, two of the species (I. *bonnali* and *I. aurelioi*) show a Z_1_Z_2_W multiple sex-chromosome system, which is very infrequent among Lacertidae (Odierna et al. 1996; Mezzasalma et al. 2021).

Female heterogamety is the only sex-chromosome system that has been found in Lacertidae (Mezzasalma et al. 2021). Except for two discordant reports involving *L. agilis* (Srikulnath et al. 2014) and *L.* *schreiberi* (Rojo 2015), all tested species appear to share homologous Z chromosomes which date back to the last common ancestor of the whole group, thus supporting the long-term stability of their ZZ/ZW chromosome systems (Rovatsos et al. 2019). The case for *L. agilis* has been strongly contested by Lisachov et al. (2020), while that for *L. schreiberi* must now be rejected on the basis of the evidence presented in this article. On the other hand, cytogenetic analyses, mainly accomplished through Giemsa staining, C-banding and G-banding (Olmo et al. 1986, 1987; Odierna et al. 1993; Rojo et al. 2014), revealed extensive variability in the morphology and degree of differentiation of the W chromosome across the family, spanning from those completely euchromatic and homomorphic with the Z, to others strongly heterochromatic and morphologically distinct. These cytological observations were complemented by fluorescence in situ hybridization (FISH) studies, whereby it was concluded that the extent of heterochromatinization of the W chromosome appears to be associated with independent, species-specific, extensive accumulation of DNA repeats. Taking all this evidence together, the W chromosome thus appears to be the most dynamic component of Lacertidae genomes (Pokorná et al. 2011; Matsubara et al. 2014b; Mezzasalma et al. 2016; Giovannotti et al. 2018; Suwala et al. 2020).

When whole genome sequences are not available, chromosome homology among species is best determined through molecular cytogenetic analyses, which can reveal the evolutionary complexity hidden under morphologically similar (using standard banding and staining techniques) chromosome patterns (Matsubara et al. 2014a). Here, we describe the preparation of flow-sorted chromosome paints from the Iberian Rock lizard *I. monticola* (BOULENGER, 1905), and exemplify their subsequent use in cross-species chromosome painting to carry out comparative analyses of chromosome evolution. There are currently nine recognized species in *Iberolacerta*, a genus almost entirely confined to small widely separated mountain areas in the Iberian Peninsula and in the Balkan Peninsula (Arribas et al. 2014). Cytogenetic surveys based on conventional staining and banding techniques showed that, except for the three Pyrenean species, *Iberolacerta* species show a similar karyotypic macrostructure: 2n = 36 acrocentric chromosomes, no microchromosomes, and different degrees of W chromosome differentiation (Odierna et al. 1996; Arribas and Odierna 2004; Arribas et al. 2006; Rojo et al. 2014). The Pyrenean species, on the other hand, displayed reduced diploid numbers and many biarmed chromosomes that probably evolved from the ancestral acrocentric chromosomal complement through a series of Robertsonian fusions (Odierna et al. 1996; Olmo et al. 2004). (Odierna et al. 1996; Arribas and Odierna 2004; Arribas et al. 2006; Rojo et al. 2014).

To assess the use of flow-sorted chromosome paints for cross-species comparative analyses, we applied the *I. monticola* chromosome paints to study the chromosome evolution in the following lacertid species: the congeneric *I. galani* (ARRIBAS, CARRANZA & ODIERNA 2006) (2n = 36), with ZW sex chromosomes (Arribas et al. 2006); I. *bonnali* (LANTZ, 1927) (2n = 24 in males, 2n = 23 in females), with 12 biarmed chromosomes and a multiple Z_1_Z_2_W chromosome system (Odierna et al. 1996); *Lacerta schreiberi* (BEDRIAGA, 1878) (2n = 38), possessing the standard Lacertini karyotype (Mateo and Cano 1991); and *Timon lepidus ibericus* (LÓPEZ-SEOANE, 1885) (2n = 36), with a metacentric chromosome pair presumably produced by fusion of two large acrocentric chromosomes, a pair of microchromosomes, and a W sex microchromosome (de Smet 1981; Olmo et al. 1987; Mateo et al. 1999). Comparison of sex chromosomes at the molecular level was further extended through comparative genomic hybridization (CGH) between *I. monticola*, *L. schreiberi* and *T. lepidus*.

## Materials and methods

### Animal samples

Two adult females and one adult male of *I. monticola* were collected from the population of the fluvial valley of the river Eume (A Coruña, Spain). The tail tips from one adult female of *L. schreiberi* and another one of *T. lepidus* were collected at the Natural Park Montes do Invernadeiro (Ourense, Spain); additionally, one adult female of *L. schreiberi* was collected at Aranga (A Coruña, Spain). Finally, two adult females of *I. galani* and the tail tip of one adult female of *I. bonnali* were collected at the localities of A Ponte, Pena Trevinca (Ourense, Spain) and Pico de Urdiceto, Pirineos (Huesca, Spain), respectively. The sex of each animal was determined by examination of sexually dimorphic external morphology. All these samples were used to make metaphase chromosome spreads. Permissions for fieldwork and ethics approval of experimental procedures were issued by the competent authorities (Xunta de Galicia, Junta de Castilla-León and Gobierno de Aragón, in Spain) in accordance with Spanish legislation (Royal Decree 1201/2005 and Law 32/2007, on the protection of animals used for experimentation and other scientific purposes). All the animal samples used in this study were generously supplied by Pedro Galán (Departamento de Bioloxía, Universidade da Coruña).

### Metaphase chromosomes preparation

The tail tip collected from each specimen (approximately 10 mm) was pre-treated before setting up the cell cultures as described in Ezaz et al. (2008), with slight modifications. Briefly, the surfaces of the tail tips were sterilized by wiping with gauze soaked in 70% ethanol, clipped and incubated at 30ºC for 24 h in Collection Medium [RPMI 1640 Medium containing 25 mM HEPES (Sigma) with 1 mg/mL kanamycin (Sigma) and 1% antibiotic-antimycotic (Life Technologies-Gibco).

Fibroblast cell lines and metaphase chromosome spreads were prepared as described in Rojo et al. (2014). Cultures for flow-sorting were split up to 4 passages before the chromosomes were harvested.

### Probe preparation, karyotyping and C-banding

Chromosome paints from a female *I. monticola* were prepared from chromosomes sorted with a dual laser cell sorter (Mo-Flo, Dako) at the Cambridge Resource Centre for Comparative Genomics, Department of Veterinary Medicine, University of Cambridge, Cambridge, UK, as previously described (Yang et al. 1995). Sorted chromosomes were used as templates for DNA amplification by DOP-PCR (Telenius et al. 1992). Primary DOP-PCR products were used as templates in a secondary DOP-PCR to incorporate biotin-16-dUTP (Roche).

TaqI sat DNA species-specific probes were prepared as described in Rojo et al. (2015).

For karyotyping, the slides were stained with DAPI (1.5 µg/mL) in anti-fade medium Vectashield (Vector Laboratories). Sequential C-banding + CMA_3_ + DAPI staining was performed as described in Rojo et al. (2014).

### Fluorescence in situ hybridization and signal detection

The chromosome content and purity of flow-sorted fractions was first determined by FISH onto metaphase spreads of female *I. monticola*. Unidirectional chromosome painting with the probe containing the W sex chromosome of *I. monticola* was performed on *I. galani*, *I. bonnali*, *L. schreiberi* and *T. lepidus*. Three additional probes were applied for the characterization of *I. bonnali*, whereas the full set of chromosome-specific probes of *I. monticola* was used in cross-species hybridization to metaphase spreads of *L. schreiberi* and *T. lepidus*.

FISH was performed using the protocols described in Yang et al. (Yang et al. 1995); Rens et al. (2006), with several modifications. Briefly, slides were dehydrated through ethanol series; aged at 65 °C for 1 h; denatured in 70% formamide/2x saline-sodium citrate (SSC) at 70 °C for 1 up to 3 min (time depending on species and metaphase preparation) and dehydrated again. One microliter of biotinylated probe was made up to 12 µL with hybridization buffer (50% deionized formamide (v/v), 10% dextran sulfate, 2x SSC, 0.05 M phosphate buffer, pH 7.3). This mixture was denatured at 75 °C for 10 min, preannealed at 37 °C for 30 min and applied to each slide. Hybridization was carried out at 37 °C overnight, for the same species, and over 48 and 72 h, for congeneric and more distantly related species, respectively. Posthybridization washes were performed in 50% formamide/2x SSC twice for 5 min each, followed by 2x SSC twice for 5 min each and 4x SSC with 0.05% Tween-20 (4xT) once for 4 min. Washes were carried out at 42 °C. Probe detection was performed using 200 µL of diluted (1:500) Cy3-Streptavidin antibody (Amersham) per slide at 37 °C for 30 min. After detection, slides were washed in 4xT three times for 3 min each at 42 °C and mounted in with anti-fade medium Vectashield (Vector Laboratories) containing 1.5 µg/mL DAPI.

### Interspecies comparative genomic hybridization (iCGH)

Total genomic DNA was extracted from ethanol preserved tissues of *I. monticola*, *L. schreiberi* and *T. lepidus* females using a commercial kit (RealPure Genomic DNA Extraction Kit, Durviz), following the manufacturer’s instructions. Total genomic DNA was labeled by random priming with the Prime-It Random Priming Labeling Kit (Agilent Technologies), according to the manufacturer’s specifications. Genomic DNAs of *I. monticola*, *L. schreiberi* and *T. lepidus* were labeled, respectively, with TRITC-dUTP, FITC-dUTP, and both TRITC-dUTP and FITC-dUTP. iCGH was performed as described in Rojo et al. (2014). Reciprocal iCGH experiments were done between each pair of species. For each slide that was made, 250 ng of TRITC-labeled and 250 ng of FITC-labeled DNA were ethanol-precipitated with 20 µg of glycogen and 4 µg of unlabeled, sheared genomic DNA (as competitor) derived from a male of the same species as the target metaphases.

### Microscopy and data analyses

At least 20 metaphase spreads were examined after each hybridization. Images were captured using the epifluorescence microscopes Leica DMRXA and Nikon Microphot-FXA, equipped with cooled CCD cameras [Photometrics Sensys and DS-Qi1Mc (Nikon Instruments), respectively]. The Leica CW4000 FISH and the NIS-Elements D 3.10 (Nikon Instruments) softwares were used to capture 16-bit grey-scale images of DAPI, Cy3/TRITC and FITC signals, which were then normalized and merged to a 24-bit colour image. For karyotyping, the DAPI images were displayed in contrast-adjusted reversed greyscale images. The final composition of the images was performed with Adobe *Photoshop* CS4 11.0.1 (Adobe Systems Inc.).

## Results

### Karyotyping and C-banding

**Fig. 1:**
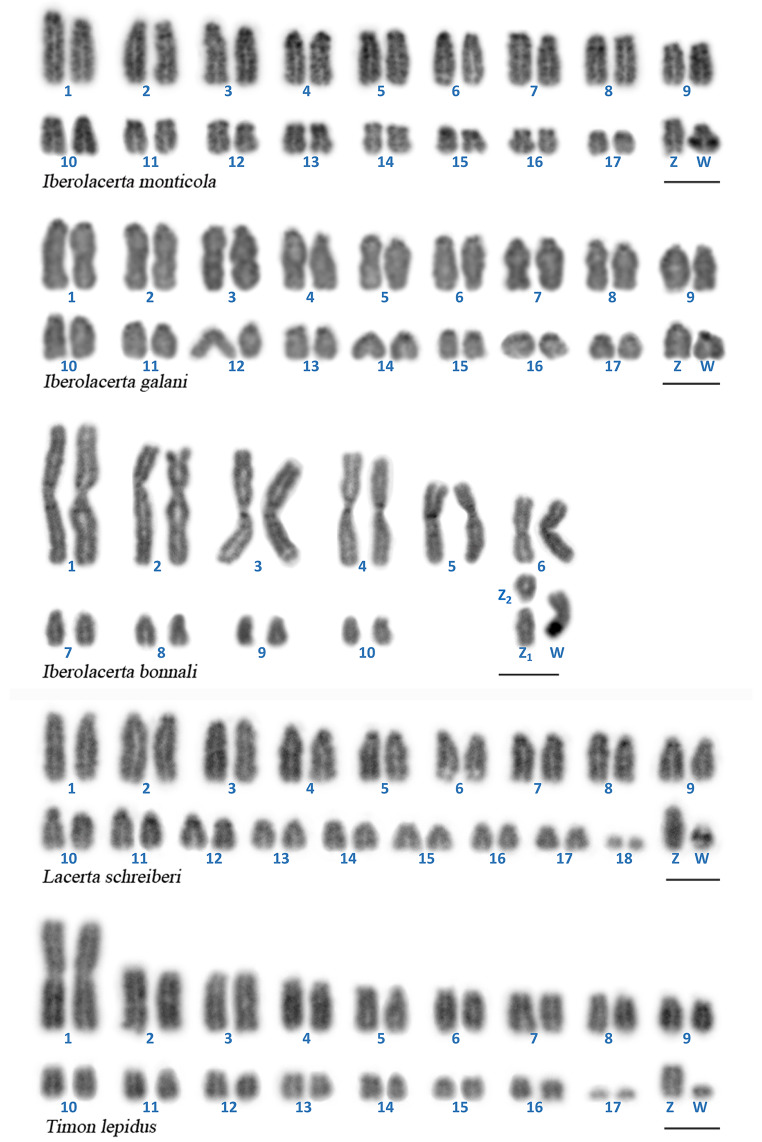
Karyotypes of female specimens of each of the species studied arranged from DAPI stained metaphases. Scale bars represent 5 μm

DAPI-stained karyotypes of all the analyzed species are shown in Fig. 1. The karyotypes of *I. monticola* and *I. galani* (2n = 36) consisted exclusively of acrocentric chromosomes of gradually decreasing size. A similar heteromorphic sex chromosome pair was found in female specimens of both species, in which the W chromosome is distinctly smaller than the Z counterpart, and showed an intense fluorescent signal after DAPI staining.

**Fig. 2:**
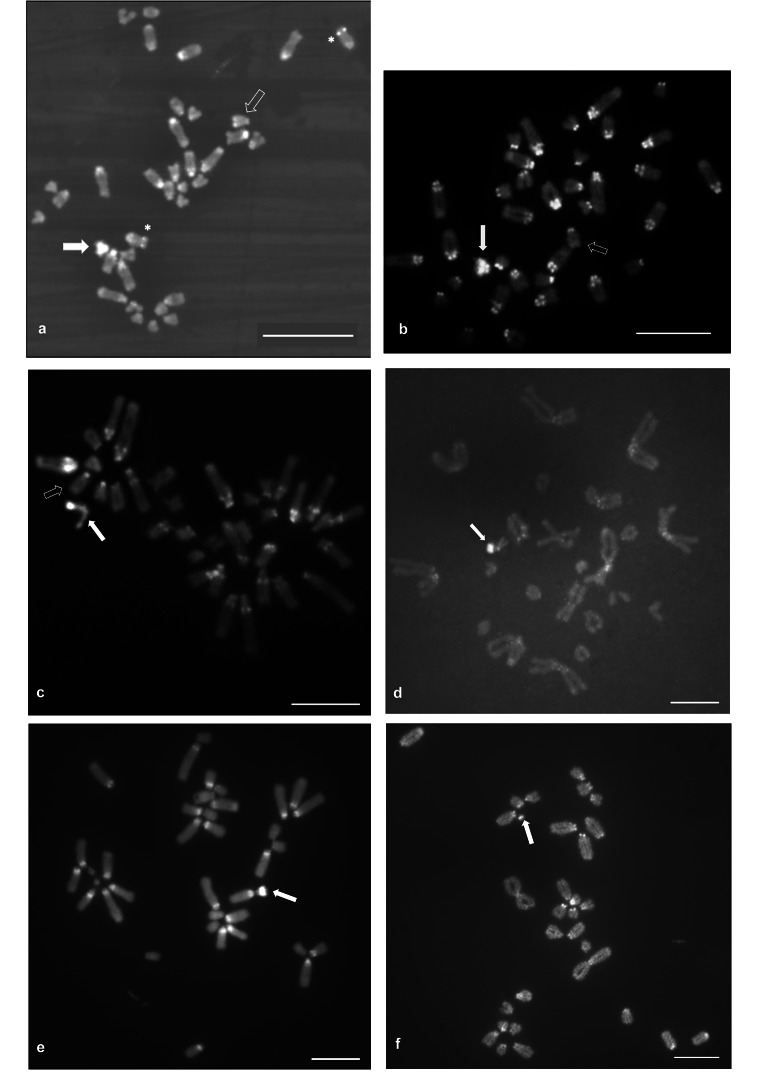
Metaphase plates of females of the different species studied in this paper. Metaphases were sequentially stained with C-banding + CMA_3_ (**a**) + DAPI (**b-f**). Species studied include *I. monticola* (**a, b**), *I. galani* (**c**), *I. bonnali* (**d**), *L. schreiberi* (**e**), and *T. lepidus* (**f**). Filled arrows point to W chromosomes, whereas empty arrows in **a**, **b** and **c** point to Z chromosomes. Asterisks in **a** indicate CMA_3_-positive signals associated with NORs in chromosome 6 of *I. monticola*. Scale bars represent 10 μm

The diplod chromosome number in the female *I. bonnali* was 2n = 23, and the karyotype comprised 13 biarmed and 10 acrocentric chromosomes. In this species, the W chromosome is a metacentric element, and its meiotic homologues —Z_1_ and Z_2_— are two smaller acrocentric elements. A bright DAPI-positive region was observed in the q arm of the W chromosome.

The karyotype of *L. schreiberi* (2n = 38) was composed of 36 acrocentric chromosomes, gradually decreasing in size, and a pair of microchromosomes. The female specimen analyzed in the first instance for this study, from the population of Invernadeiro, showed a markedly heteromorphic pair formed by a very small, DAPI-positive element, and a medium-sized counterpart, tentatively identified as the Z in this species.

The karyotype of female *T. lepidus* (2n = 36) contained one large metacentric chromosome pair, 32 acrocentric chromosomes and two microchromosomes. The smallest acrocentric chromosome, barely larger than the microchromome pair, was distinctively stained by DAPI and it is most likely to be the W sex chromosome, while the putative Z was identified as a medium-sized acrocentric element.

C-banding revealed similarities in the abundance and distribution of constitutive heterochromatin in the karyotypes of these species, such as the presence of DAPI- and CMA_3_-positive centromeric and interstitial/pericentromeric blocks, and the occurrence of GC-rich, faint telomeric C-bands in at least the largest chromosomes of the karyotypes (Fig. 2). Differences in the C-banding patterns of these species were mainly associated to the sex chromosomes. The W chromosomes of *I. monticola* and *I. galani* are almost completely heterochromatic, with only a small euchromatic region located in an interstitial position (Fig. 2a–c). There is considerable heterogeneity in size and overall appearance of this chromosome among different metaphases, apparently brought about by differences in the extent of DNA denaturation and loss produced by the C-banding pretreatment of the preparations (see also Arribas et al. 2006; Rojo et al. 2014). The submetacentric W chromosome of *I. bonnali* shows a prominent C-band in the distal region of the q-arm (Fig. 2d). In *L. schreiberi*, the smallest chromosome of the heteromorphic pair (the putative W chromosome) is also easily recognizable after C-banding by bearing a prominent heterochromatin block in interstitial position (Fig. 2e). This same pattern is found in the W chromosome of *T. lepidus*, which, despite its small size, seems to be only partially heterochromatic with an interstitial C-positive region surrounded by proximal and distal euchromatic areas (Fig. 2f). In all the cases, the heterochromatin of the W chromosomes resulted intensely stained after both DAPI and CMA_3_ staining. On the other hand, the Z chromosome of *I. monticola* and *I. galani* could be distinguished from the autosomes by bearing a brighter, CMA_3_-positive telomeric C-band, which is most clearly shown before DAPI staining (Fig. 2a–c).

### Flow sorting of I. monticola chromosomes and characterization of painting probes

**Fig. 3:**
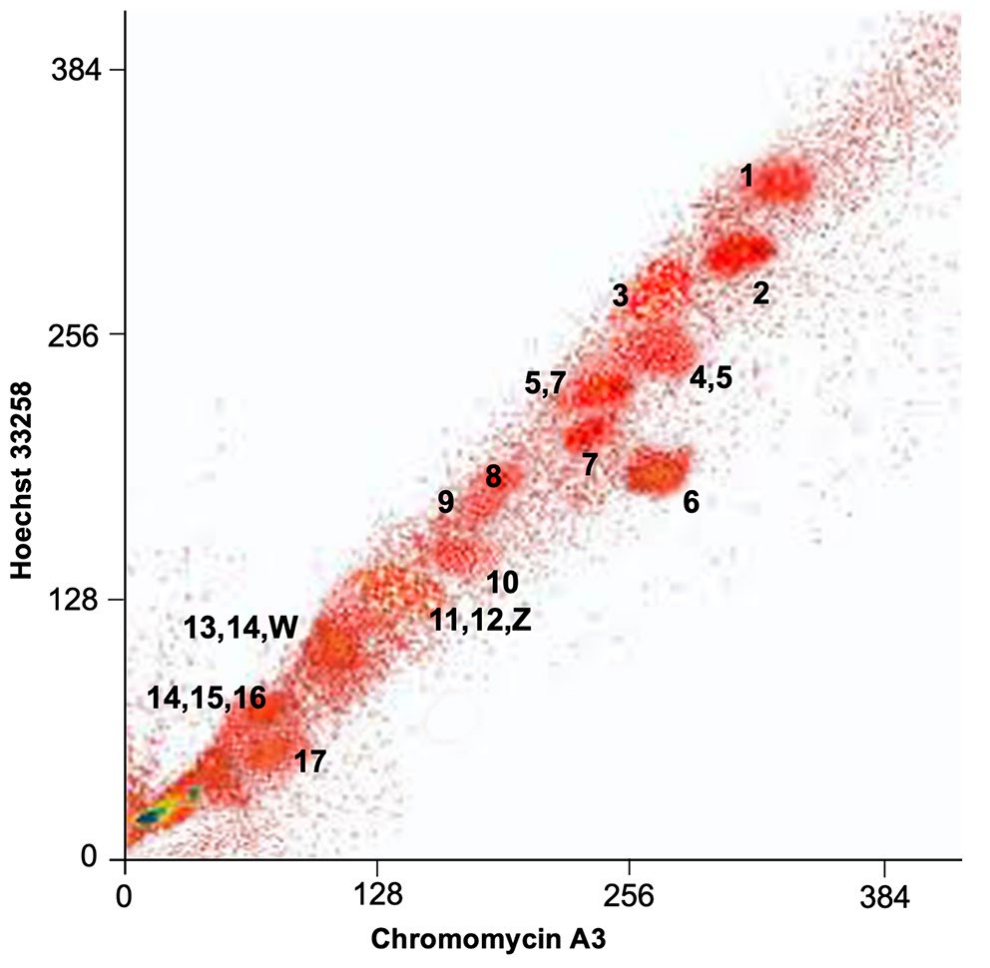
Flow-sorted karyotype of *Iberolacerta* monticola. The x and y axes report fluorescence intensity for the corresponding fluorochrome. The painting probes derived from the different separate flow peaks are indicated

The 36 chromosomes of the karyotype of *I. monticola* were differentiated into 14 separate flow peaks (Fig. 3). Painting probes (pp) from each peak were hybridized onto *I. monticola* metaphase chromosomes to determine the chromosome content of these flow peaks (Fig. S1, Supplementary Information). Nine chromosome pairs were resolved separately, which provided chromosome-specific painting probes (pp1–pp3, pp6–pp10, and pp17). In addition, two peaks contained two chromosomes each (pp4,5 and pp5,7), and three peaks contained three chromosomes each (pp11,12,Z, pp13,14,W, and pp14,15,16). The presence of the same chromosome in adjacent flow peaks, as it is the case with chromosomes 5, 7 and 14 (see Fig. 3) could be an indication of the two homologues differing in their repetitive DNA content, but the close similarity to the sizes and DAPI banding patterns of other chromosomes of the karyotype preclude our exploration of this interesting possibility with the available data.

### Cross-species chromosome painting

**Fig. 4:**
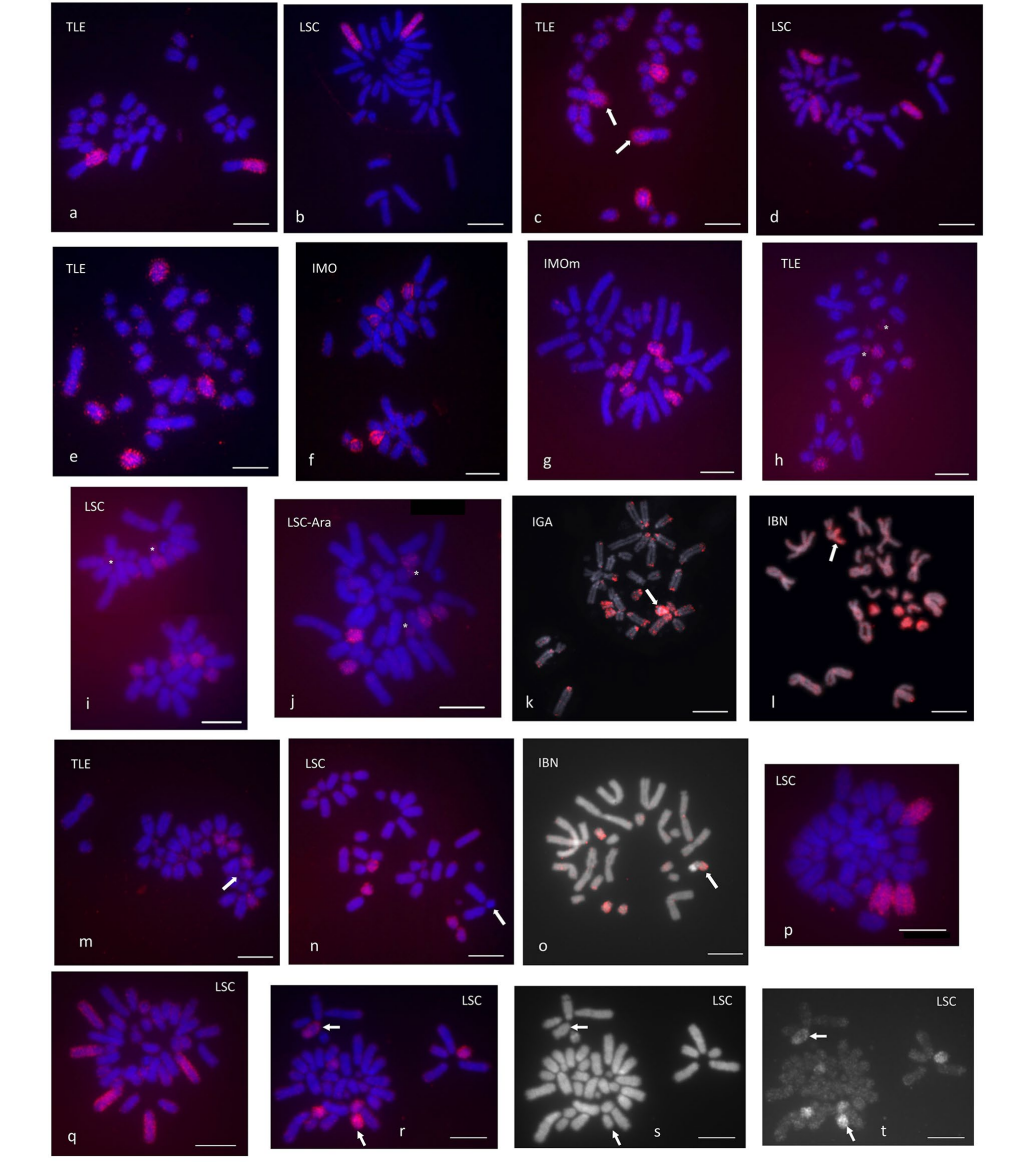
Cross-species chromosome painting of metaphase plates with different *I. monticola* flow-sorted chromosome probes (pp, see Fig. 3). All but one of the hybridizations (panel g)correspond to female specimens. IBN, *I. bonnali*; IGA, *I. galani*; IMO, *I. monticola*; IMOm, *I. monticola male*; LSC, *L. schreiberi* Invernadeiro; LSC-Ara, *L. schreiberi* Aranga; TLE, *T. lepidus.* Scale bars represent 10 μm. **a, b**: pp2 on TLE and LSC; **c, d**: pp4,5 on TLE (arrows point to the p-arm of chromosome 1) and LSC; **e**: pp 5,7 on TLE; **f–j**: pp11,12,Z on IMO, IMOm, TLE, LSC, and LSC-Ara (asterisks mark the microchromosome pair); **k–n**: pp13,14,W on IGA, IBN, TLE and LSC (the arrow points to the W chromosome of each species); **o**: pp14,15,16 on IBN (the arrow points to the p-arm of the neo-W chromosome); **p**: trisomy 3, pp3 on LSC; **q**: trisomy 5, pp4,5 on LSC; **r–t**: segmental duplication 16, pp14,15,16 on LSC (**r**, merged; **s**, DAPI; **t**, CY3; arrows point to the heteromorphic pair)

The study of chromosome synteny with the whole set of *I. monticola* probes on *L. schreiberi* and *T. lepidus* revealed a high degree of karyotype conservation between the three species (see Figs. S2 and S3, Supplementary Information, for the complete results of chromosome painting on these species). Most *I. monticola* chromosomes were completely preserved —both in DNA content and morphology— in the other lacertids. One of the few rearrangements detected involved *I. monticola* chromosomes 2 and 4, which correspond to the q and p-arms of the metacentric chromosome 1 of *T. lepidus*, respectively (Fig. 4a, c), while being homologous to acrocentric chromosomes 2 and 4 of *L. schreiberi* (Fig. 4b, d). Arrows in Fig. 4c point to the p-arm of *T. lepidus* chromosome 1, which was painted by the pp4,5 probe, but not by pp5,7 (Fig. 4e).

The probe pp11,12,Z painted an odd number of medium-sized chromosomes in *I. monticola* (Fig. 4f). The unpaired chromosome —which, according to its size, could be the 11th largest chromosome— is presumably the Z sex chromosome. Chromosome painting with this probe on male *I. monticola* metaphases labeled an even number of chromosomes, thus confirming that this flow peak contains the Z chromosome (Fig. 4g). Similarly, pp11,12,Z hybridized to five medium-sized acrocentric chromosomes on female *T. lepidus* and *L. schreiberi* (two populations) metaphases, strongly suggesting the structural conservation of the Z, which could be the tenth and ninth largest element of the karyotype, respectively (Figs. 1 and 6 h–j). In addition, this probe clearly marked the microchromosome pair in both species (asterisks in Fig. 6 h–j), thus indicating that these elements were most likely fused to either chromosomes 11, 12 or Z in the last common ancestor of *Iberolacerta* species.

The probe pp13,14,W, containing the *I. monticola* W sex chromosome together with autosomes 13 and 14, hybridized to the euchromatin of the W chromosome in *I. galani* (Fig. 4k), and to the euchromatin at the end of the q-arm of the submetacentric W chromosome in *I. bonnali* (Fig. 4l). It also painted two small, acrocentric chromosome pairs in both species. A screening with the remaining flow-sorted fractions of *I. monticola* showed that the p-arm of the W chromosome of *I. bonnali* was only marked by the probe pp14,15,16 (Fig. 4o), indicating that it must be homologous to either autosome 15 or 16 of *I. monticola*. When the probe pp13,14,W was used on *T. lepidus* and *L. schreiberi*, it painted a pair of small acrocentric chromosomes in each species (12 and 13 in *T. lepidus*; 14 and 15 in *L. schreiberi*), but no signal was detected on the W chromosome of either species (Fig. 4m, n).

In the screening of metaphase plates to determine the hybridization results described above, some metaphases with chromosomal mutations, such as trisomies (Fig. 4p, q) or segmental duplications (Fig. 4r-t), were detected, apparently produced during the culture of the fibroblast cell lines.

### Interspecies comparative genomic hybridization (iCGH) and FISH with a satDNA probe

**Fig. 5:**
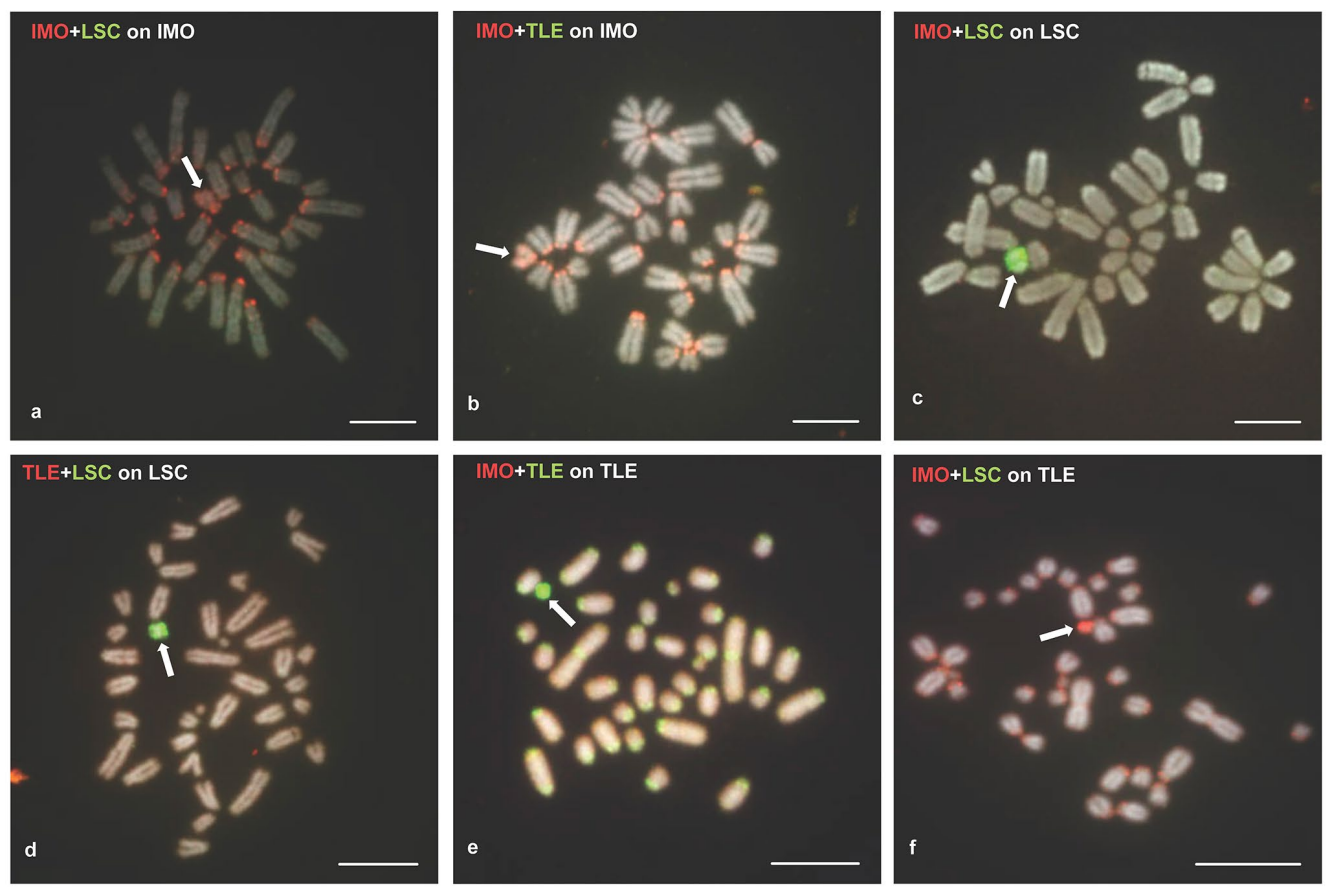
Interspecies comparative genomic hybridization on female metaphases of *I. monticola* (**a**, **b**), *L. schreiberi* (**c**, **d**), and *T. lepidus* (**e**, **f**). Genomic DNA of *I. monticola* is stained with TRITC (IMO; red), genomic DNA of *L. shcreiberi* is stained with FITC (LSC; green), and genomic DNA of *T. lepidus* is stained with both FITC (**b**, **e**) and TRITC (**d**, **f**). Arrows point to W chromosomes. Scale bars represent 10 μm

**Fig. 6:**
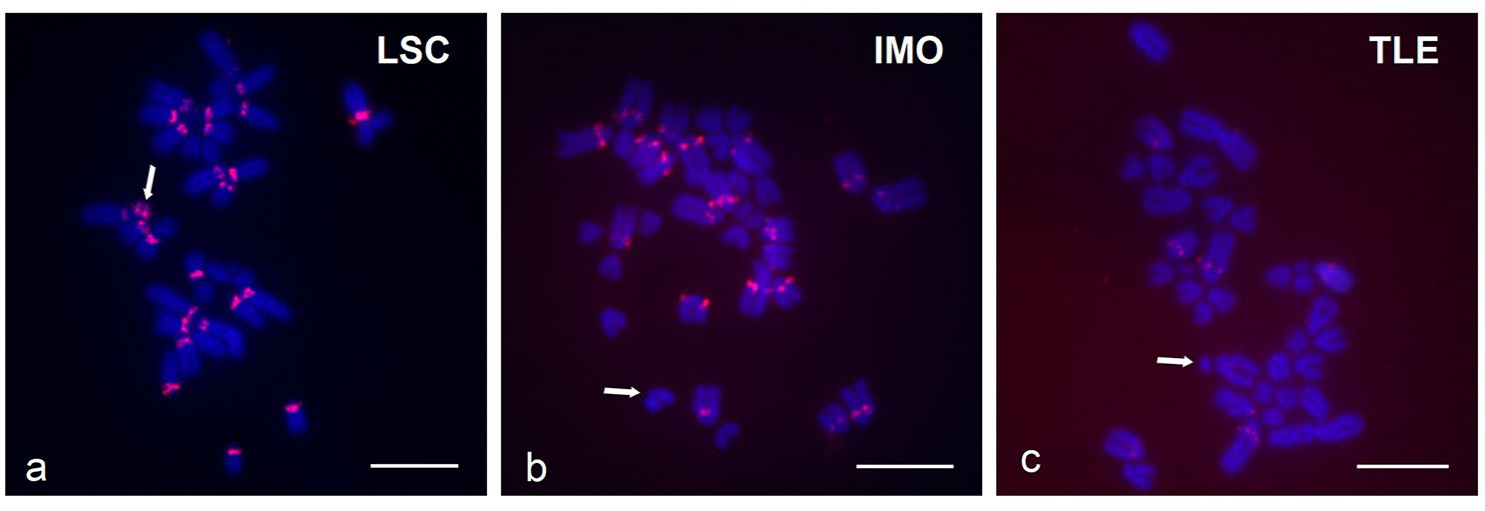
Hybridization pattern of the TaqI satellite DNA probe on female metaphase spreads of *L. schreiberi* (**a**), *I. monticola* (**b**), and *T. lepidus* (**c**). Arrows point to W chromosomes. Scale bars represent 10 μm

Absence of hybridization signal with the pp13,14,W probe on the W chromosomes of *L. schreiberi* and *T. lepidus* led us to further investigate the differentiation of W chromosomes among the three species by carrying out iCGH. Reciprocal iCGH experiments highlighted the accumulation of species-specific sequences in the chromosomes previously identified as the W chromosome of each species (Fig. 5). For instance, the W chromosome of *I. monticola* was predominantly labeled by *I. monticola* genomic DNA when co-hybridized with genomic DNA of either *L. schreiberi* or *T. lepidus* (Fig. 5a, b). The same pattern was observed in metaphases of *L. schreiberi* and *T. lepidus* (Fig. 5c, d, e and f, respectively). Due to the bright signals produced by the repetitive content of the W chromosomes, it was not possible to elucidate if the molecular composition of sex chromosomes differed only at the heterochromatic or also at the euchromatic regions. Additional evidence on the nucleotide divergence of W chromosomes was obtained after FISH with a satellite DNA probe, TaqI, which showed that although this satellite family is dispersed over several chromosomes of the karyotype of these three species, only the W chromosome of *L. schreiberi* harbored repeats (Fig. 6).

## Discussion

Four main conclusions of evolutionary relevance can be drawn from the results of this work. The first refers to microchromosomes, or rather their absence. Squamates show a marked tendency towards reduction in the number of microchromosomes through lineage-specific fusions of microchromosomes to different macrochromosomes, and a concomitant increase in G-banding patterns, which may have a direct influence on recombination levels and chromosome mutation rates (Olmo 2008; Srikulnath et al. 2021). This trend is particularly enhanced in Gekkota (Srikulnath et al. 2015) and in lacertid lizards, whose karyotypes generally show only one pair of microchromosomes and, in a few species, their complete disappearance (e.g., *Zootoca vivipara* and *Atlantolacerta andreanskyi*) (Olmo et al. 1991; Olmo and Signorino 2005). The lack of microchromosomes in the genus *Iberolacerta* is therefore atypical among Lacertidae. We have determined that the ancestral microchromosomes in the *I. monticola* karyotype are most likely fused to either the Z sex chromosome or to one of the autosomes labelled as 11 and 12. The possibility of the hybridization signals on the *L. schreiberi* and *T. lepidus* microchromosomes being due to repetitive centromeric DNA shared by any of the *I. monticola* chromosomes in the pp11,12,Z is quite improbable. In previous works, we found that the HindIII satDNA repeats overlapped the centromeric heterochromatic blocks of all the chromosomes of the *I. monticola* karyotype (Giovannotti et al. 2014). Therefore, if the hybridization signals from the microchromosomes were due to the presence of centromeric repeats, we should have detected their signals with all our probes. Considering how rare the absence of microchromosomes is in Lacertidae karyotypes, and since all the species of the genus *Iberolacerta* share this trait, it seems logical to assume that the fusion observed in *I. monticola* most likely constitutes a synapomorphy for *Iberolacerta* within the Lacertidae, i.e., a shared derived trait that should have been already present in the last common ancestor of this small species group. Phylogenetic reconstructions indicate that *Iberolacerta* originated 22–30 mya, together with many other genera of Lacertini (Hipsley et al. 2009, additional file 1; Garcia-Porta et al. 2019, supplementary Fig. 11), well before the Pleistocene glacial oscillations, with its now-recognizable species having been preserved in glacial refugia in Southern European mountains, thanks to its complex topography and history, that allowed their allopatric divergence (Carranza et al. 2004; Crochet et al. 2004; Arribas et al. 2014). Population fragmentation was probably enhanced during the Messinian Salinity Crisis (5.9–5.3 Mya), when the nearly complete desiccation of the Mediterranean Sea forced the retreat of mesic species, such as *Iberolacerta* spp., to the moister Atlantic-influenced areas and to the mountainous regions around the Mediterranean Basin. The same climatic events were experienced in much the same way by *Atlantolacerta andreanskyi* (Werner, 1929), another lacertid species showing independent microchromosome loss. This species is a member of the subtribe Eremiadini endemic to the High Atlas Mountains in northern Africa, where their highly fragmented populations in different mountaintops could actually harbor at least six well differentiated species (Barata et al. 2012). Similarly to *Iberolacerta* and *Atlantolacerta*, microchromosomes have been independently lost in *Zootoca vivipara* (Lichtenstein, 1823), the terrestrial reptile with the largest geographical and highest latitudinal distribution, inhabiting quite different biogeographic regions in the Northern Hemisphere (IUCN 2022), but exhibiting similar responses to glaciations than species from temperate zones (Horreo et al. 2018). Perhaps the most important ecological characteristic that all these three taxa have in common is their independent conquest of cold environments (Garcia-Porta et al. 2019). Taking into consideration the evolutionary importance attributed to microchromosomes (Uno et al. 2012; Deakin and Ezaz 2019; Srikulnath et al. 2021; Waters et al. 2021) and the apparently direct involvement of some of their gene contents in the differential adaptations of other sqamates (Bentley et al. 2023), we cannot discard the possibility that their loss has been due to selection. In fact, the loss of microchromosomes in three independent lineages may represent a case of evolutionary convergence.

The second *a priori* evolutionary relevant result reported in this paper refers to the origin of the Z_1_Z_2_W multiple sex-chromosome system in two of the three Pyrenean species of *Iberolacerta*, *I. bonnali* and *I. aurelioi*. DNA phylogenies have not been able to resolve the splitting order of these species, probably because the time interval between their speciation events was too short (Mayer and Arribas 2003; Arribas et al. 2014), but allozymes (Mayer and Arribas 1996), and both karyological (Odierna et al. 1996) and osteological data (Arribas 1998) suggest that *I. aranica* has most likely diverged first, before the rearrangement that gave rise to the multiple sex chromosome system, which would represent a derived character shared by *I. bonnali* and *I. aurelioi*. According to our results, that rearrangement included a fusion between the ancestral W and one of the autosomes belonging to either pair 15 or 16, which gave rise to a biarmed neo-W, whereas the homologous chromosome in that pair became the Z_2_. This is interesting because W-autosome fusions or translocations are not the only possible ways to produce a Z_1_Z_2_W sex chromosome system. In insects, for example, it is far more common for such complex sex chromosomes to appear due to Z fissions rather than W fusions with an autosome (Blackmon et al. 2016). In addition, the evolutionary outcome of a W-autosome fusion could be analogous to other rearrangements that restrict recombination on the sex specific chromosome, assuming that the fused autosome contains sexually antagonistic variation (Charlesworth and Charlesworth 1980). However, in contrast to many families of Iguania, where multiple sex chromosomes are extraordinarily frequent, the sex-chromosome rearrangements in Lacertidae appear to be evolutionary dead ends, since only two other species besides *I. bonnali* and *I. aurelioi* are known to present them, namely *Podarcis tauricus* and *Z. vivipara* (Mezzasalma et al. 2021).

The third conclusion of our research is that the previously reported independent origin of the Z chromosome in *L. schreiberi* (Rojo 2015) is not correct. Quite on the contrary, the results of chromosome painting on this species, as well as on all the others studied in this paper, are consistent with the conservation of Z chromosome homology, in agreement with prior reports on the long-term stability of the Z chromosome in lacertid lizards (Rovatsos et al. 2019; Lisachov et al. 2020). We must conclude, therefore, that the previous observations apparently supporting that claim of the independent origin of the Z in this species were artifacts, produced by chromosomal aberrations, which most likely arose spontaneously during fibroblast culture. In addition to the Z chromosome, the autosomes showed an overall conservation of syntenic relationships among *Iberolacerta* spp., *T. lepidus* and *L. schreiberi*, with centric fusions between acrocentric chromosomes giving rise to the new biarmed metacentric chromosomes, and no insertional translocations having been ever observed.

Finally, regarding the evolution of nucleotide sequences in the ancestral W chromosome of *Iberolacerta*, it is clear that their divergence among *I. monticola*, *I. galani* and *I. bonnali* is not as large as to preclude their hybridization with the *I. monticola* probe. On the contrary, since this probe did not paint the W chromosomes of either *T. lepidus* or *L. schreiberi*, we must conclude that this is due to the high divergence among their nucleotide sequences. The results from iCGH and FISH with a satDNA probe in these species confirmed that the W chromosome is the fastest evolving element of the karyotype, and that its evolution is at least in part due to satellite DNA turnovers, as it has been repeatedly observed in other comparative analyses of lacertid lizards (Matsubara et al. 2015; Giovannotti et al. 2018; Suwala et al. 2020).

### Electronic supplementary material

Below is the link to the electronic supplementary material.

Supplementary Material 1
