# Supplementary material for: Chromosome evolution in Iberolacerta, a genus that deviates from the standard karyotype formula of Lacertidae
Source: Genetica. 2023 Sep 1;151(4-5):267–79. doi: 10.1007/s10709-023-00194-w (PMC10654178; doi:10.1007/s10709-023-00194-w)
Supplement: Supplementary file 1 — Supplementary Material 1 [file 10709_2023_194_MOESM1_ESM.pdf]

Supplementary information

Chromosome evolution in a genus that deviates from the standard karyotype formula of Lacertidae

Fig. S1. Chromosomal assignment of flow karyotype peaks on female I. monticola. Arrows in **k** and **l** point to the Z and W sex chromosomes, respectively. Scale bars = 10 µm.

Fig. S2. Chromosome painting with the whole set of I. monticola probes on female L. schreiberi. Arrow in **l** points to the W chromosome. Scale bars = 10 µm.

Fig. S3. Chromosome painting with the whole set of I. monticola probes on female T. lepidus. Arrow in **l** points to the W chromosome. Scale bars = 10 µm.

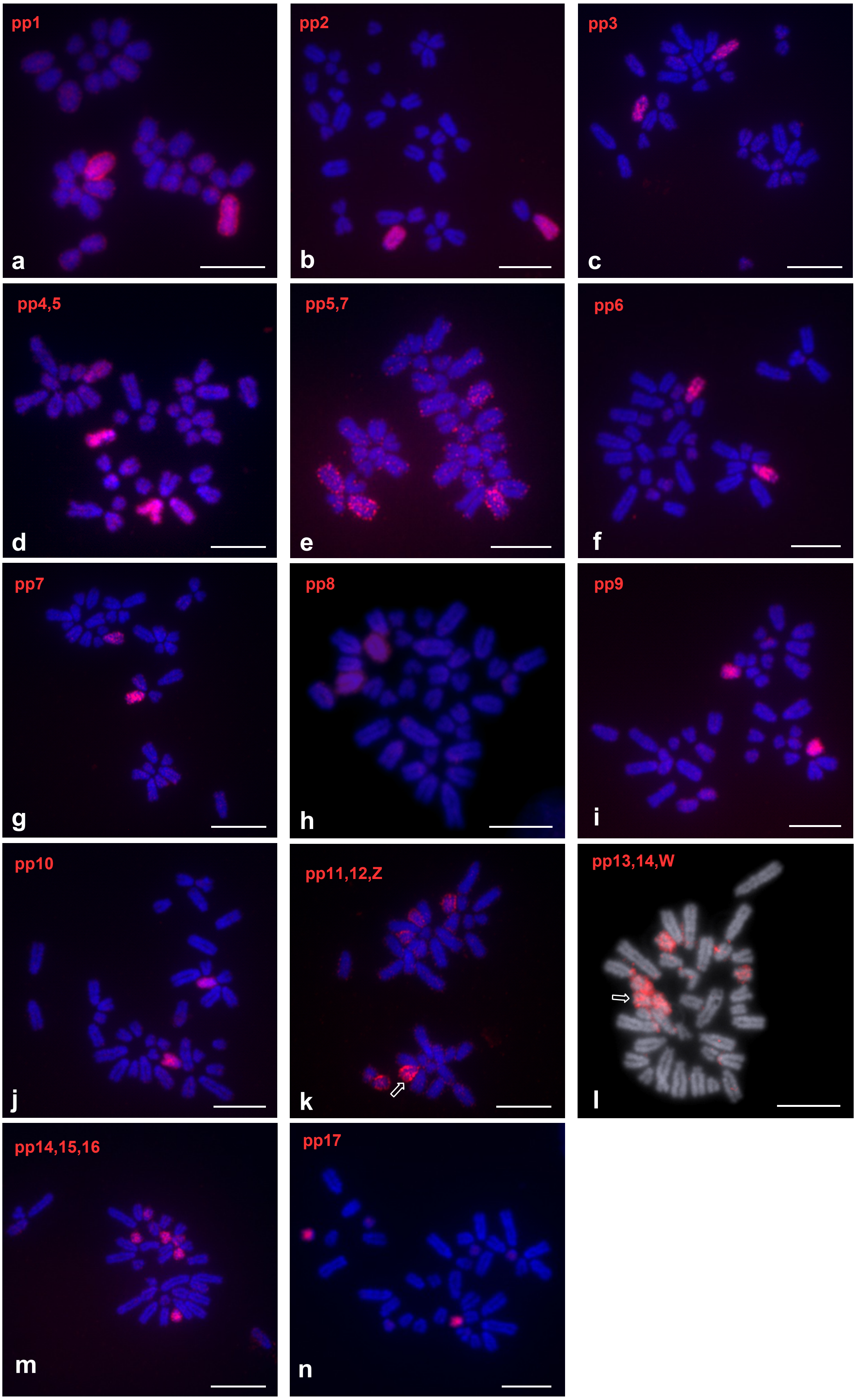
pp1
a
pp2
b
pp3
c
pp4,5
d
pp5,7
e
pp6
f
pp7
g
pp8
h
pp9
i
pp10
j
pp11,12,Z
k
pp13,14,W
l
pp14,15,16
m
pp17
n

Figure S1

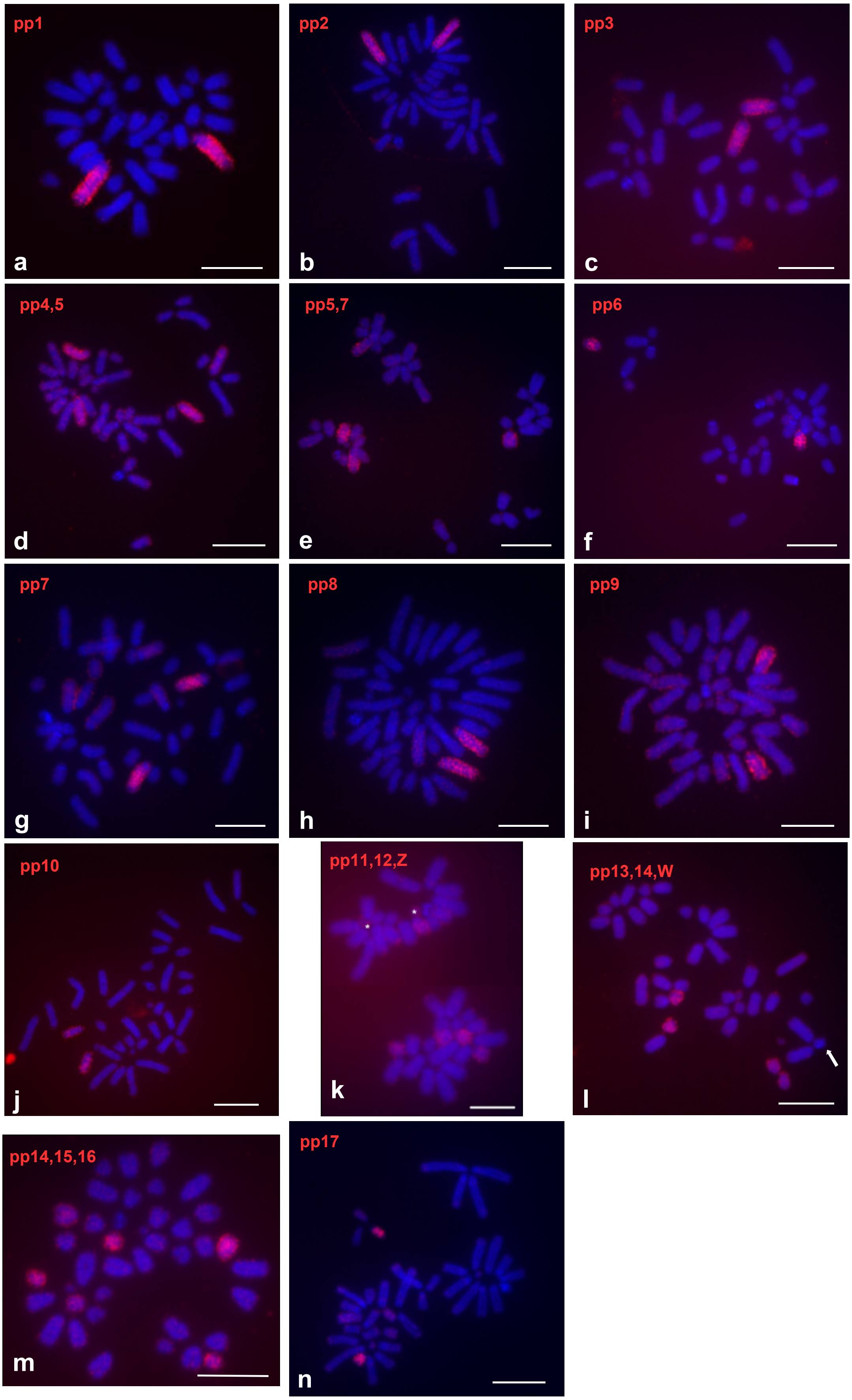

pp1
a
pp2
b
pp3
c
pp4,5
d
pp5,7
e
pp6
f
pp7
g
pp8
h
pp9
i
pp10
j
pp11,12,Z
k
pp13,14,W
l
pp14,15,16
m
pp17
n

Figure S2

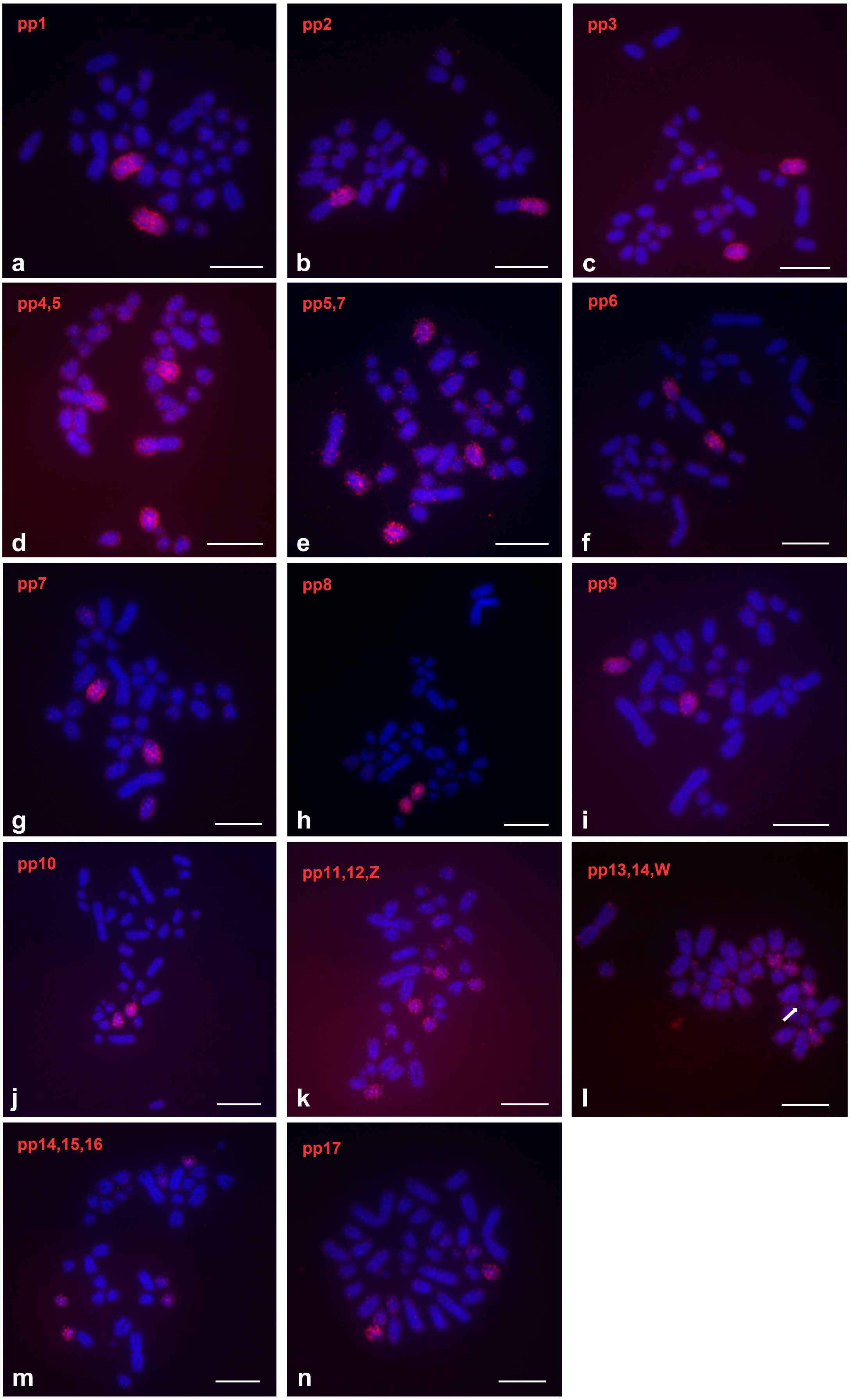
pp1
a
pp2
b
pp3
c
pp4,5
d
pp5,7
e
pp6
f
pp7
g
pp8
h
pp9
i
pp10
j
pp11,12,Z
k
pp13,14,W
l
pp14,15,16
m
pp17
n

Figure S3
